# Application of Micro-Percutaneous Nephrolithotomy (Micro-PCNL) combined with FURL in 1-2 cm symptomatic, refractory lower calyceal stones

**DOI:** 10.12669/pjms.39.6.6782

**Published:** 2023

**Authors:** Xiao-qiang Shi, Tao Ma, Wentao Wang, Zhenyu Cui, Xin-yao Li, Ying Gao

**Affiliations:** 1 Xiao-qiang Shi, Department of Urology, Affiliated Hospital of Hebei University, Baoding 071000, Hebei, China; 2 Tao Ma, Department of Urology, Affiliated Hospital of Hebei University, Baoding 071000, Hebei, China; 3 Wentao Wang, Department of Urology, Affiliated Hospital of Hebei University, Baoding 071000, Hebei, China; 4 Zhenyu Cui, Department of Urology, Affiliated Hospital of Hebei University, Baoding 071000, Hebei, China; 5 Xin-yao Li, Hebei University School of Basic Medical Sciences, Shijiazhuang 050013, Hebei, China; 6 Ying Gao, Department of Urology, Affiliated Hospital of Hebei University, Baoding 071000, Hebei, China

**Keywords:** Micro-Percutaneous Nephrolithotomy, Flexible ureteroscope, Combined therapy, Lower calyceal stones

## Abstract

**Objective:**

To investigate the safety and efficacy of Micro-Percutaneous Nephrolithotomy (Micro-PCNL) combined with flexible ureteroscopic lithotomy (FURL) in the treatment of 1-2 cm symptomatic, refractory lower calyceal stones.

**Methods:**

A retrospective analysis was performed concerning the clinical data of 28 patients with 1-2 cm symptomatic, refractory lower calyceal stones. When there was a difficulty in performing FURL in Affiliated Hospital of Hebei University from January 2019 to February 2022, ultrasound-guided F4.8 visual puncture was performed on the lower calyceal stone,with a holmium laser was then used to treat the remaining stones, followed by drainage using a flexible ureteroscopic sheath and postoperative indwelling of the ureteral stent without a nephrostomy tube. The surgery time, intraoperative bleeding and stone-free rate(SFR) were recorded, and the VAS score was used to evaluate the patients’ pain status.

**Results:**

The surgery was completed successfully in an average of 43.46 ± 10.04 minutes, and the puncture time was 3.46 ± 0.69 minutes. The SFR was 85.71%(24/28) and 92.86%(26/28) at one day and 30 days after surgery, respectively. Two patients with residual stones greater than 0.6 cm in size underwent extracorporeal shock wave lithotripsy four weeks after surgery. Patients were followed up for three months after surgery, and the SFR was revised to 96.43%(27/28). In addition, the VAS scores of all patients decreased significantly from before to after surgery, and the difference was statistically significant(p< 0.05).

**Conclusion:**

Micro-Percutaneous Nephrolithotomy (Micro-PCNL) combined with FURL is safe and effective in the treatment of 1-2 cm symptomatic, refractory lower calyceal stones.

## INTRODUCTION

Extracorporeal shock wave lithotripsy(ESWL), flexible ureteroscopic lithotomy (FURL) and percutaneous nephrolithotomy (PCNL) are common choices for the treatment of lower calyceal stones. ESWL is preferred when the stone diameter is <1 cm, and PCNL is preferred for stones >1-2 cm in size. However, the use of PCNL in the treatment of lower calyceal stones 1-2 cm in size remains controversial.^1^,^2^ Due to their complex structure, lower calyceal stones have several problems, such as a low stone-free rate (SFR) and difficult stone removal with FURL during the treatment when compared with other types of renal stones. Micro-Percutaneous Nephrolithotomy refers to PCNL under needle-like visualised nephroscopy, which is a new method of minimally invasive treatment for lower calyceal stones.^3^

However, a single application of Micro-Percutaneous Nephrolithotomy to treat lower calyceal stones leads to the risk of high intrapelvic pressure due to the lack of sheath and washing fluid drainage. Meanwhile, when applying transurethral FURL alone to treat 1-2 cm lower calyceal stones, in the case of a limited infundibulopelvic angle (IPA), the ureteroscope may not reach the lower calyx, resulting in intraoperative difficulty with lithotripsy. Accordingly, in our study, Micro-Percutaneous Nephrolithotomy combined with FURL was used for the treatment of 1-2 cm symptomatic, refractory lower calyceal stones and good results were achieved.

## METHODS

This is a retrospective analysis. A total of 28 patients with 1-2 cm symptomatic, refractory lower calyceal stones of (1.63 ± 0.26 cm on average) treated in the Affiliated Hospital of Hebei University from January 2019 to February 2022.It included 18 males and 10 females aged from 24 to 76 years, with an average age of 57.50 ± 12.49 years. All patients had unilateral kidney stones, including 16 cases with stones located in the left kidney and 12 cases in the right kidney. There were six cases of multiple stones and 22 of single stones; the stones ranged from 1.0 to 2.0 cm in size, with an average size of 1.63 ± 0.26 cm (Table-I). Twenty-one patients had a medical history of undergoing ESWL. All cases were complicated with macroscopic haematuria, urinary tract infection, pain in the affected side of the kidney and other symptoms to varying degrees.

**Table-I:** General data of the included patients.

Age (years)	Stone size (cm)	Stone location		Number of stones	

		Left kidney (%)	Right kidney (%)	Single (%)	Multiple (%)
57.50 ± 12.49	1.63 ± 0.26	16 (57.14)	12 (42.86)	22 (78.57)	6 (21.43)

### Etical Approval

This study was approved by the Institutional Ethics Committee of the Affiliated Hospital of Hebei University(No: HDFY-LL-2018-003) on January 2, 2018; the consent taken in retrospect after discharging the patient.

### Inclusion criteria:

- Patients with lower renal calyceal stones 1.0-2.0 cm in size diagnosed by imaging examinations such as intravenous urography (IVU), urinary system ultrasonography (USG) and CT.
- Patients with clinical symptoms such as renal pain, haematuria etc. and patients with single or multiple lower calyceal stones.

### Exclusion criteria:

- Patients with abnormal renal anatomy, such as UPJ obstruction, medullary sponge kidney, polycystic kidney, horseshoe kidney etc.
- Patients with blood system diseases and abnormal coagulation function.
- Patients who were taking anticoagulant drugs such as aspirin, warfarin, dabigatran, rivaroxaban, apixaban and other patients without drug withdrawal for over two weeks.
- Patients with fever or urinary tract infection not treated according to the inclusion criteria.
- Patients with preoperative abnormal renal function (endogenous creatinine clearance rate < 50 ml/min).
- Patients with moderate and severe hydronephrosis (separation of the collecting system > 20 mm by renal colour ultrasound).
- Pregnant women and menstruating females.
- Patients who could not tolerate anaesthesia or surgery due to serious systemic diseases, heart diseases, pulmonary insufficiency or serious organ failure.

All patients underwent USG, IVU and CT before surgery to determine the size and location of the stones. Routine blood and urine tests, blood biochemistry, ECG and other examinations were completed to exclude surgical contraindications. Patients with urinary tract infection indicated by a routine urine test received a urine culture and a drug susceptibility test, and the appropriate antibiotics were used to treat the infection; prophylactic antibiotics were used 30 minutes. before surgery.

Under general anaesthesia, the patient’s affected side was raised by 30º-35º to maintain the oblique supine lithotomy position. After a routine disinfection towel was applied, the guide wire was first placed into the renal pelvis under the ureteroscope, and then the guide sheath of the F12/14 flexible ureteroscope was placed along the guide wire. After that, the ureteroscope was placed to locate the stones. A 200 μm holmium laser fibre was inserted into the channel of the flexible ureteroscope for lithotripsy.

In the case of difficult lithotripsy, the position closest to the target renal calyces in the area between the inferior edge of the 12th rib, the posterior axillary line and the scapular line was selected as the puncture site. Under ultrasound guidance, the visual puncture system was used for PCNL into the lower calyces of the kidney. With the assembly of an F4.8 visualised nephroscope, a 200 μm holmium laser was placed for lithotripsy of lower calyceal stones under the nephroscope. Drainage was continued using a flexible ureteroscopic sheath, and some stone fragments were removed with a reticular basket under the ureteroscope. No nephrostomy tube was left after surgery, and the F5 double ‘J’ tube was left indwelling in the ureter and urinary catheter.

On the first day at 9:00am after surgery, KUB or CT were performed again to understand the status of lithotripsy, stone discharge and the position of the double ‘J’ tube. When there were residual stones (>4 mm), postural therapy was administered postoperatively for auxiliary lithotripsy. If there were no obvious complications, the patients were discharged from the hospital on postoperative day one after the removal of the urinary catheter. The patients were reviewed one month after surgery, and the double ‘J’ tube was removed as appropriate. The stone removal was observed three months after surgery. Assessment of efficacy: VAS scores were recorded before and one day, 30 days and three months after surgery to evaluate the improvement of pain before and after surgery. According to a postoperative review based on KUB or CT, stone removal was identified when patients had no residual stones or the residual stones were <4 mm in size. The procedure was done by the surgeon group.

## RESULTS

The surgery was completed successfully in an average of 43.46 ± 10.04 minutes (32-67 minutes), and the visual puncture time was 2-4 minutes, with an average of 3.46 ± 0.69 minutes. The haemoglobin decreased by 0.6-1.2 g/L at one d after surgery, with an average of 0.95 ± 0.17 g/L. One case developed a fever, which was improved by symptomatic anti-inflammatory treatment. The postoperative hospital stay was 1-3 days, with an average of 1.50 ± 0.64 days. The SFR was 85.71% (24/28) and 92.86% (26/28) at one day and 30 days after surgery, respectively. Two patients with residual stones greater than 0.6 cm in size underwent ESWL four weeks after surgery. Patients were followed up for three months after surgery and the SFR was revised to 96.43%, as shown in Table-II.

**Table-II:** Surgery and hospitalisation of patients.

Surgery time (min)	Visual puncture time (min)	Length of stay in hospital (d)	Decrease in haemoglobin 1d after surgery (g/L)	Stone clearance		

				1day (%)	30 days (%)	3 months (%)
43.46 ± 10.04	3.46 ± 0.69	1.50 ± 0.64	0.95 ± 0.17	24 (85.71)	26 (92.86)	27(96.43)

All patients were observed, and no cases of delayed bleeding or serious infection occurred during treatment and follow-up. In addition, there was a statistically significant difference in the VAS score of patients at one day, 30 days and three months after surgery when compared with that before treatment (p< 0.05). All patients’ pain was essentially relieved at 30 days and three months after surgery, and the difference in VAS score was statistically significant compared with that at 1d after surgery (p< 0.05), as shown in Table-III.

**Table-III:** Comparison of preoperative and postoperative VAS scores.

	Time points			

	Before surgery	1 day after surgery	30 days after surgery	3 months after surgery
VAS score	7.57 ± 0.50	3.46 ± 0.79*	0.14 ± 0.36^Δ^	0.04 ± 0.19^Δ^

***Note:***

* The difference was statistically significant when compared with that before surgery (p< 0.05);

Δ The difference was statistically significant when compared with that 1 d after surgery (p< 0.05).

## DISCUSSION

In this study, a combined therapy using needle-like visual nephroscope-guided PCNL was applied simultaneously for the treatment of symptomatic refractory lower calyceal stones 1-2 cm in size that could not be managed with a flexible ureteroscope. Consequently, there was a relatively low SFR postoperatively, with only a few mild complications and a short hospital stay, which confirmed the safety and effectiveness of needle-like visual nephroscope-guided PCNL combined with FURL.This is consistent with the research conclusion of Chinese scholars.^4^

Although percutaneous nephrolithotripsy is the gold standard for treating large stones^5^, it has the advantages of short surgical time and high stone clearance rate. However, the incidence of complications and decreased hemoglobin levels during percutaneous nephrolithotomy were significantly higher than those during FURL when the stones were greater than 2 cm.^6^ Due to certain limitations in the curvature of the end of the flexible ureteroscope, especially when a holmium laser fiber is placed in the working channel, the sensitivity will decrease, and there is an infundibulopelvic angle (IPA) in the lower renal calices. Ureteroscopic treatment of lower renal calices stones has a high residual rate of stones^7^, especially when the IPA angle is less than 30^o^ the stone clearance rate significantly decreases.^8^

The treatment effect of a single retrograde ureteroscopic surgery for 1-2 cm lower renal caliceal stones is not ideal. Moreover, Jeong et al.^9^ proposed that the stone clearance rate of percutaneous nephroscopy is higher than that of ureteroscopy for the treatment of lower calyceal stones. Therefore, we used retrograde ureteroscopic lithotripsy combined with ultrasound-guided F4.8 visual puncture system ultramicro channel percutaneous nephrolithotripsy to improve the stone clearance rate of lower renal calyx stones.

The reason why standard channel and microchannel percutaneous nephroscopy for the treatment of lower renal calyx stones is prone to bleeding and injury is that when the target renal calyx is the lower calyx, the channel pathway is longer. The F4.8 visual puncture system guided by ultrasound is more slender and has dual guiding and positioning functions. The puncture channel is monitored by ultrasound and conducted in areas with low blood flow. During the puncture process, various anatomical levels can be observed to avoid damage to blood vessels and surrounding organs. Stop the puncture after observing the stones and proceed with lithotripsy. In line with the concept of precision surgery, it can accurately reach the stone site, which is particularly important for patients with small caliceal stones in the anterior group of the lower calyx.

The stones in this area are not easy to find and are considered an independent risk factor for residual stones, avoiding damage caused by excessive puncture depth.^10^ Bader et al.^11^ employed a needle-like visualised nephroscope for a visualised puncture during standard PCNL for the first time in 2011 and believed that this procedure could improve the safety of PCNL. In the same year, Desai et al.^12^ used Micro-Percutaneous Nephrolithotomy for the first time, and the corresponding results supported its safety, efficacy and hence, feasibility in practice. A needle-like visualised nephroscope can facilitate puncture under the dual visual conditions of ultrasound and an ultra-fine camera of the visual system, offering real-time monitoring of the puncture at the anatomical level. In this way, it can effectively avoid blood vessels and important tissues so as to reduce the impact of the invasiveness of PCNL, which can be a disadvantage during Microperc.^13^ Wicaksono F et al. have concluded that micro-PCNL is superior to FURL in managing pediatric kidney stones, 10-20 mm in size based on their comparable SFR and fewer requirements of additional stenting procedures.^14^

Through this study,we summarizes the advantages of this surgery: (1) Visual puncture ultra-micro channel nephroscopy can use different guidance methods at different anatomical levels, making the display of puncture paths clearer and more accurate, reducing channel related complications and bleeding^15^; (2) The combination of dual mirrors reduces the dead angle of the renal calyx field of view and increases the stone clearance rate; (3) Visual puncture technology has a short Learning curve and is easy to master; (4) The simple operation space of the lower renal calyx is small, and a single retrograde soft mirror surgery or a single percutaneous nephroscopy surgery can easily lead to high pressure inside the calyx.^16^ This method can reduce the pressure inside the calyx, effectively reducing the “flying” of stone debris, and providing a clearer field of vision.

### Limitations of this study

The study was a retrospective study with a relatively small sample size. Further prospective, large-scale randomised controlled studies are required to confirm the findings of our study.

## CONCLUSIONS

Micro-Percutaneous Nephrolithotomy combined with FURL is safe and effective for the treatment of symptomatic refractory lower calyceal stones 1-2 cm in size, offering the advantages of minimal trauma, accurate and effective surgery, a low rate of postoperative complications etc.

### Authors’ Contributions:

**XS** and **WW:** Designed this study, prepared this manuscript, are responsible and accountable for the accuracy and integrity of the work.

**ZC** and **TM:** Collected and analysed the clinical data.

**XL** and **YG:** Data analysis and **s**ignificantly revised this manuscript.
